# Direct anterior versus lateral approaches for clinical outcomes after total hip arthroplasty: a meta-analysis

**DOI:** 10.1186/s13018-019-1095-z

**Published:** 2019-02-26

**Authors:** Zhao Wang, Hong-wei Bao, Jing-zhao Hou

**Affiliations:** Department of Orthopaedics, Jingjiang People’s Hospital, 28 No, Zhongzhou Road, Jingjiang, Taizhou City, 214500 Jiangsu Province China

**Keywords:** Anterior approach, Lateral approach, Total hip arthroplasty, Meta-analysis

## Abstract

**Objective:**

To compare the outcomes of the direct anterior approach (DAA) with the lateral approach (LA) for total hip arthroplasty (THA) patients.

**Methods:**

Three English databases, PubMed, Embase, and the Cochrane Library, were searched for randomized controlled trials (RCTs) comparing the DAA with LA for THA. Information on the country, sample size, intervention, outcomes, and follow-up were extracted. Meta-analysis was performed using Stata 12.0.

**Results:**

Five RCTs totaling 475 patients (DAA = 236, LA = 239) were included in this meta-analysis. Compared with the LA, the DAA was associated with a reduction in the VAS at 6 weeks (weighted mean difference (WMD) = − 0.41, 95% confidence interval (CI) − 0.63 to − 0.19, *P* = 0.000) and total blood loss for THA patients (WMD = − 45.73, 95% CI − 84.72 to − 6.02, *P* = 0.024). Moreover, the DAA was associated with an increase in walking velocity (WMD = 5.01, 95% CI 2.32 to 7.70, *P* = 0.000), stride length (WMD = 3.12, 95% CI 2.42 to 3.82, *P* = 0.000), and step length (WMD = 4.09, 95% CI 1.03 to 7.14, *P* = 0.009) compared with the LA group. There was no significant difference between groups in the Harris hip score, operation time, transfusion rate, length of hospital stay, and the occurrence of complications.

**Conclusion:**

Current evidence demonstrated a trend showing that the DAA had a better effect on pain relief and blood-saving effects for THA patients. However, considering the number and sample size of the included trials, more large-scale RCTs with high quality are needed to confirm our conclusion.

## Introduction

Total hip arthroplasty (THA) is generally considered one of the most successful orthopedic surgical procedures for relieving pain, restoring hip function, and improving quality of life in patients with osteoarthritis (OA) [1, 2]. Enhancement in THA has led to faster functional recovery, shorter length of hospital stay, and higher patient satisfaction [3]. Among these factors, different surgical approaches can also affect the clinical outcomes after THA [4]. The lateral approach (LA) is the preferred procedure of approximately 42% of orthopedic surgeons worldwide [5]. However, the lateral approach requires muscle splitting, and thus, the postoperative pain is more severe [6].

The direct anterior approach (DAA) is an alternative surgical approach for THA. The DAA is less commonly used, although it is gaining popularity in recent years [7]. Advocates of the DAA suggested that the DAA is an intermuscular and internervous approach with less muscle and soft-tissue dissection [8]. Conversely, surgeons who favor the LA cite advantages of extensile exposure with low rates of postoperative instability [9]. Several randomized controlled trials (RCTs) have compared the DAA to the lateral approach for THA. Many of these studies contained relatively small samples and demonstrated inconsistent outcomes [7]. This uncertainty leaves the determination of which surgical approach to adopt to the preference of the surgeons. Mjaaland et al. [10] reported that the DAA caused less pain but higher postoperative levels of creatine kinase.

Two meta-analyses were recently published on this topic. Yue et al. [11] conducted a meta-analysis and their final conclusion was that there is a lack of sufficient evidence to conclude whether the DAA or lateral approach is superior for THA patients. In this meta-analysis, the authors included non-RCTs and did not perform subgroup analysis. Therefore, there was a large heterogeneity in their meta-analysis. Putananon et al. [12] conducted a network meta-analysis that compared the DAA, lateral, posterior, and posterior-2 approaches in THA. The results showed that the DAA and lateral approach ranked first and second, respectively, for THA. Network meta-analysis is an indirect analysis, and the evidence level was less than for direct meta-analysis.

Several more recent RCTs on this subject have been published without conclusive results. Thus, we undertook a further meta-analysis to evaluate whether the DAA is superior to the LA with respect to (1) Harris hip score; (2) pain score at 2 weeks, 6 weeks, and 12 weeks; (3) operation outcomes; and (4) complications and gait characteristics (velocity, stride length, and step length). We hypothesized that the DAA results in lower pain scores and less blood loss without increasing complications.

## Material and methods

The systematic review and meta-analysis was in accordance with the PRISMA (Preferred Reporting Items for Systematic Reviews and Meta-Analyses) guidelines [13] and AMSTAR (Assessing the methodological quality of systematic reviews) Guidelines.

### Literature search

Three English databases, PubMed, Embase, and the Cochrane Library, were searched from inception to October 10, 2018, with the following search terms: “direct anterior approach” OR “anterior” OR “direct anterior” AND “lateral approach” OR “lateral” AND “total hip arthroplasty” OR “total hip replacement” OR “THA” OR “THR” OR ““Arthroplasty, Replacement, Hip ”[Mesh]”. There was no language or publication date restriction. We also searched reference lists of included trials and related meta-analyses to identify potentially omitted studies.

### Inclusion criteria

1. Participants: patients suffered from osteoarthritis and femur head necrosis and prepared for primary THA
2. Interventions: the intervention group received the DAA for THA
3. Comparisons: the control group received LA for THA
4. Outcomes: Harris hip score at final follow-up; VAS at 2 weeks, 6 weeks, and 12 weeks; operation time; total blood loss; transfusion rate; length of hospital stay; complications; and temporal and spatial gait characteristics (velocity, stride length, and step length). Included studies should include at least one of the above outcomes.
5. Study design: RCTs were regarded as eligible for the study

### Study selection

Study selection was conducted by two reviewers. We removed the duplicates using Endnote X7 software (Thompson Reuters, CA, USA). According to the inclusion and exclusion criteria, we selected the included studies and downloaded the full text for data extraction. Discrepancies were reconciled through discussion or consultation with the author.

### Data extraction

Data in the included trials were extracted by two independent investigators. Disagreement between the two reviewers was settled by discussion and consultation with a third reviewer. The extracted information included (1) the basic characteristics of the included studies, including the authors, publication year, no. in the DAA and LA groups, mean age, female patients, BMI; (2) outcomes (Harris hip score at final follow-up; VAS at 2 weeks, 6 weeks, and 12 weeks; operation time, total blood loss, transfusion rate, length of hospital stay, and complications.); and (3) follow-up.

### Assessment of methodological quality

Two authors assessed the quality of included studies independently with the risk-of-bias assessment tool outlined in the Cochrane Handbook. Seven domains were evaluated: (1) random sequence generation, (2) allocation concealment, (3) blinding of patients and personal information, (4) blinding of outcome assessment, (5) incomplete outcome data, (6) selective reporting of risk, and (7) other biases.

### Statistical analysis

Stata 12.0 (Stata Corp., College Station, TX) was used for statistical analysis, and a *P* value < 0.05 was considered statistically significant. The risk ratio (RR) with 95% confidence intervals (CI) for the DAA compared with the LA was calculated for the transfusion rate and the occurrence of complications. Weighted mean differences (WMD) and 95% CI were calculated for continuous variables. A fixed effects model was chosen when there was no statistical evidence of heterogeneity (*I*^2^ < 50%), and a random effects model was adopted if significant heterogeneity was found (*I*^2^ ≥ 50%). In addition, publication bias was assessed by funnel plots.

## Results

### Study characteristics

**Fig. 1:**
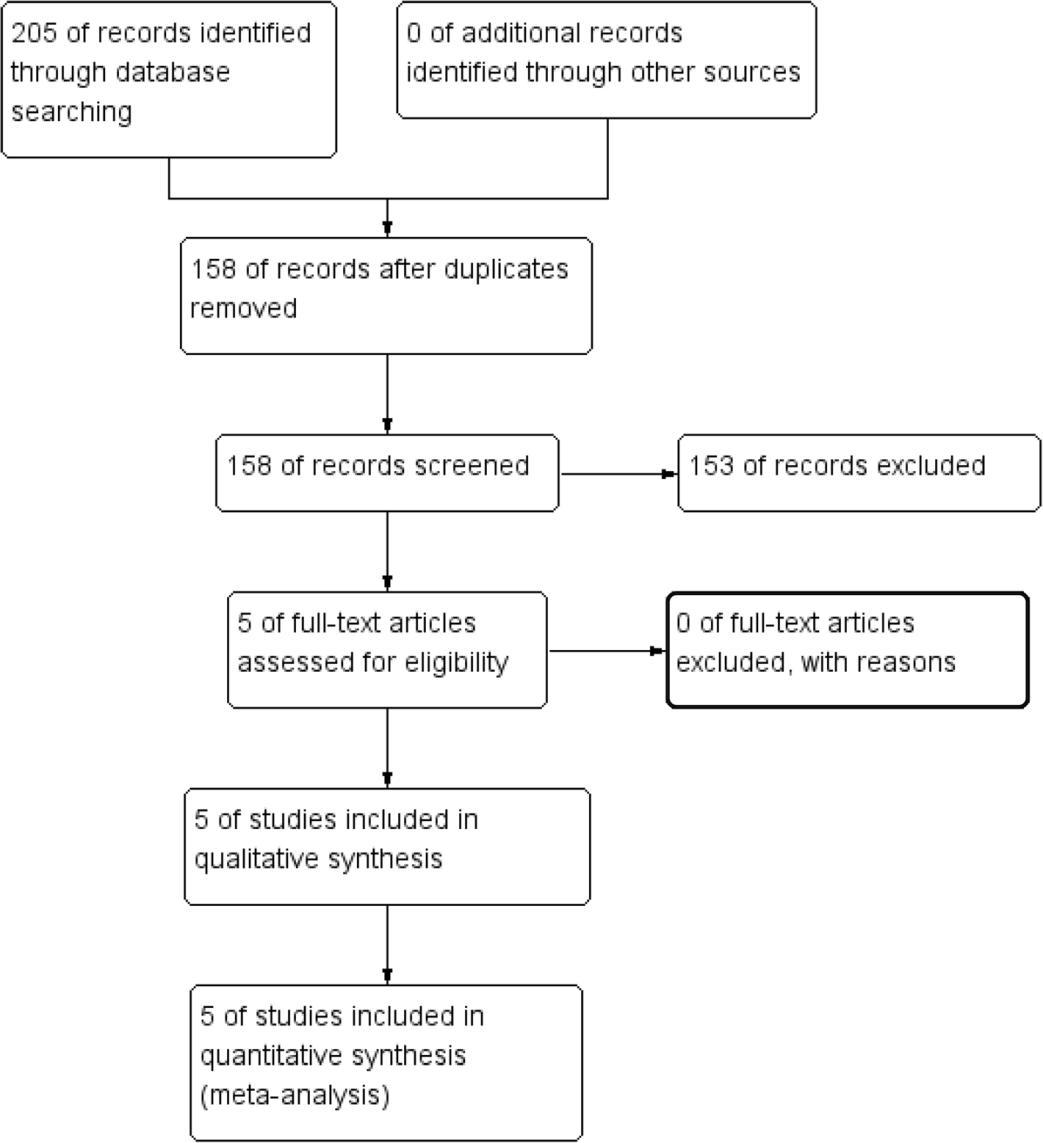
The flow diagram of study selection

The process of study selection is shown in Fig. 1. Initially, we identified 205 records through database searching. After removing duplicates, only 158 papers remained for the next step. According to the inclusion criteria, 153 records were excluded. Finally, a total of 5 RCTs [10, 14–17] met the inclusion criteria and were included in this meta-analysis.

Table 1 displays the detailed general characteristics of the included studies. A total of 475 patients were available for meta-analysis. The five eligible RCTs involved 236 hips that underwent the DAA and 239 hips that underwent the LA. All papers were published from 2009 to 2018. The sample size ranged from 16 to 84. The follow-up duration ranged from 3 to 48 months.

**Table 1:** General characteristic of the included studies. 1, Harris hip score at final follow-up; 2, VAS at 2 weeks, 6 weeks, and 12 weeks; 3, operation time, 4, total blood loss, 5, transfusion rate, 6, length of hospital stay, 7, complications, 8, temporal and spatial gait characteristics (velocity, stride length, and step length)

Author	Country	No. of patients (*n*)	Mean age (years)	Female (%)	BMI (kg/m^2^)	Outcomes	Study	Follow-up
Mjaaland 2015	Norway	84/80	66.9	66	27.65	1, 2, 3, 5, 7, 8	RCT	At discharge
Restrepo 2010	USA	50/50	67.2	69.8	27.6	1, 3, 4, 6, 9	RCT	48 months
Parvizi 2016	USA	50/50	72.4	62	28	1, 2, 5, 7, 8	RCT	12 months
Mayr 2009	Austria	16/17	66	52.5	25.6	2, 3, 4, 5, 9	RCT	3 months
Zomar 2018	Canada	36/42	60.2	52	27.9	1, 2, 3, 4, 5	RCT	3 months

### Risk of bias

**Fig. 2:**
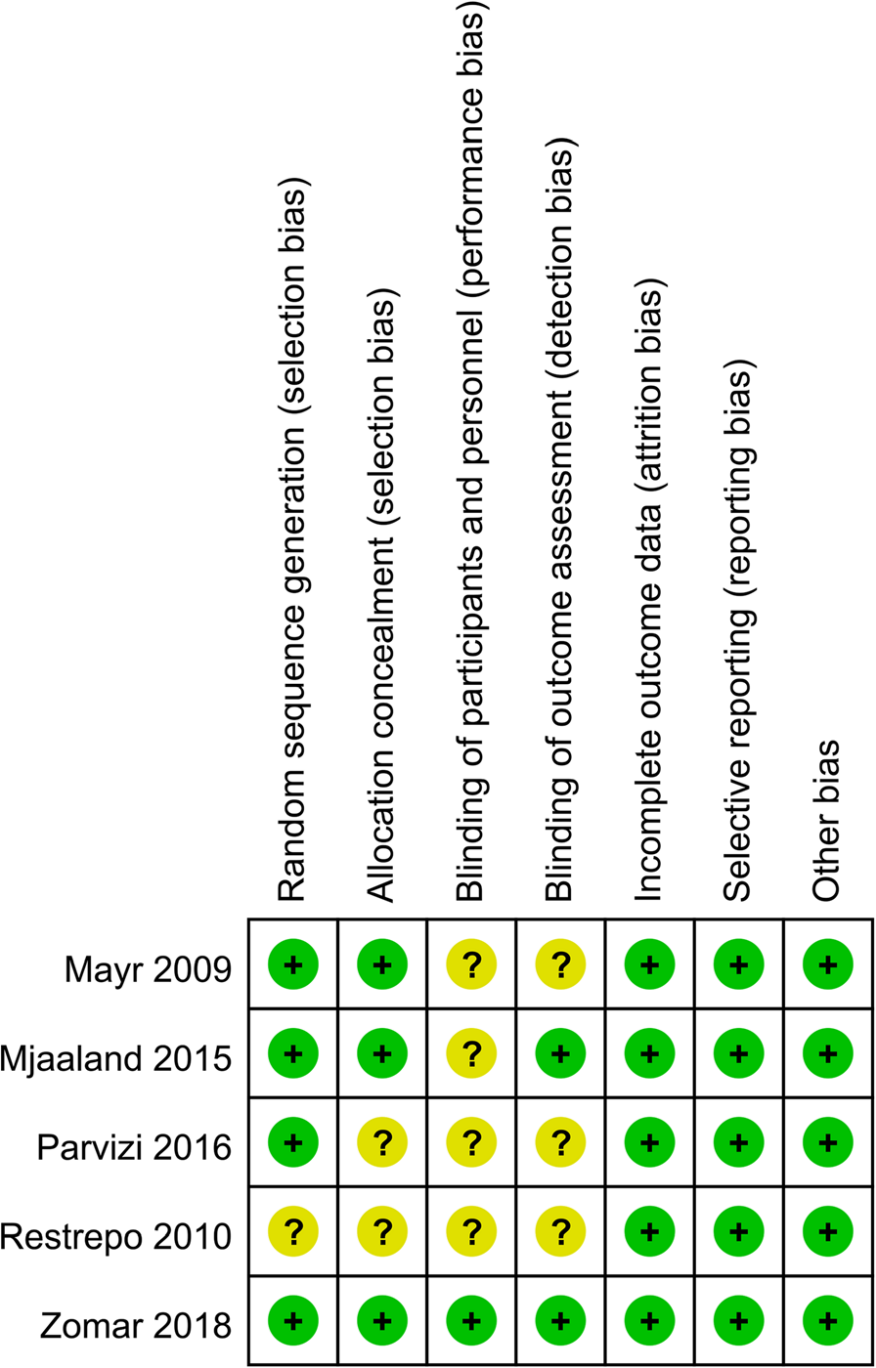
Risk of bias summary for included studies. +, no bias; −, bias;?, bias unknown

**Fig. 3:**
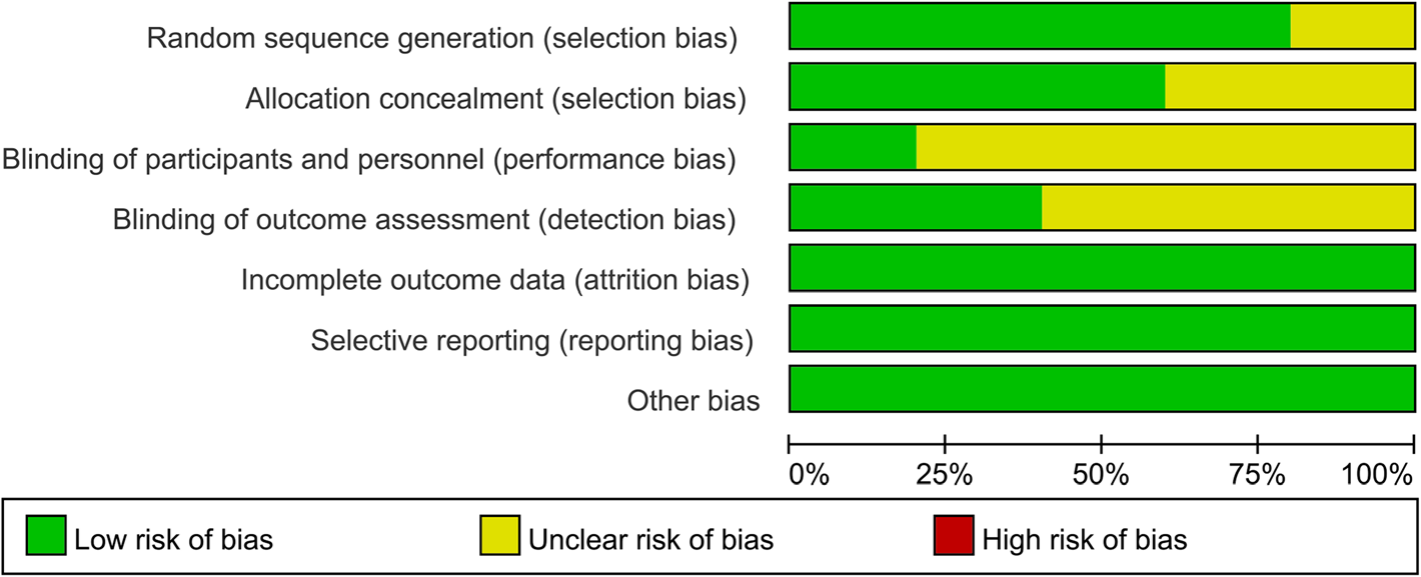
Risk of bias graph: review authors’ judgments about each risk of bias item presented as percentages across all included studies

The risk-of-bias summary and risk-of-bias graph are displayed in Figs. 2 and 3, respectively. The random sequence generation was described fairly well in four studies, and allocation concealment was described in three studies. In the remainder, this information was absent or unclear. Attrition bias, reporting bias, and other biases were all described fairly well and listed as a low risk of bias.

### Primary outcomes

#### Harris hip score at final follow-up

**Fig. 4:**
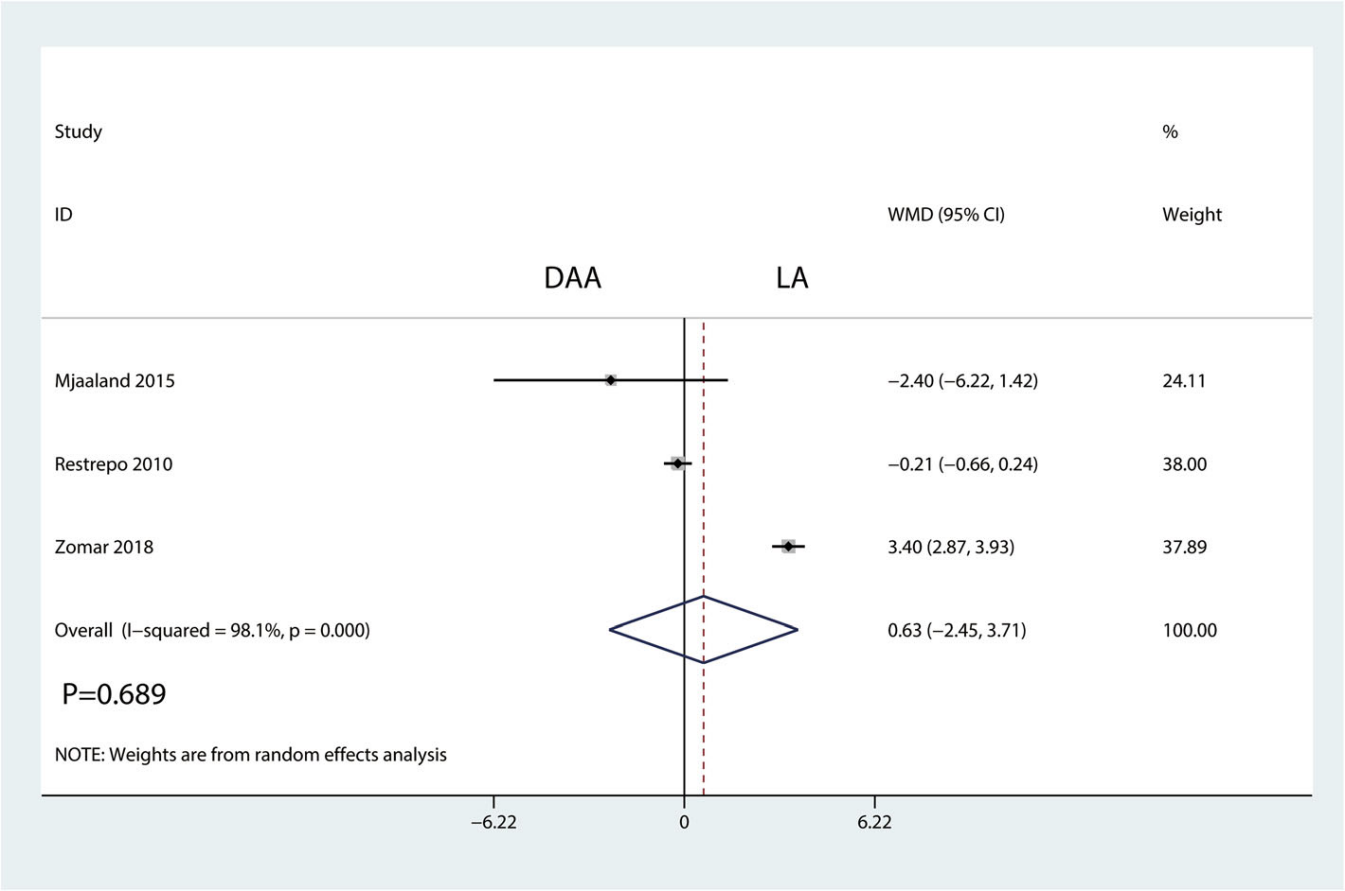
Forest plot for comparing the DAA versus LA in terms of Harris hip score at final follow-up

**Fig. 5:**
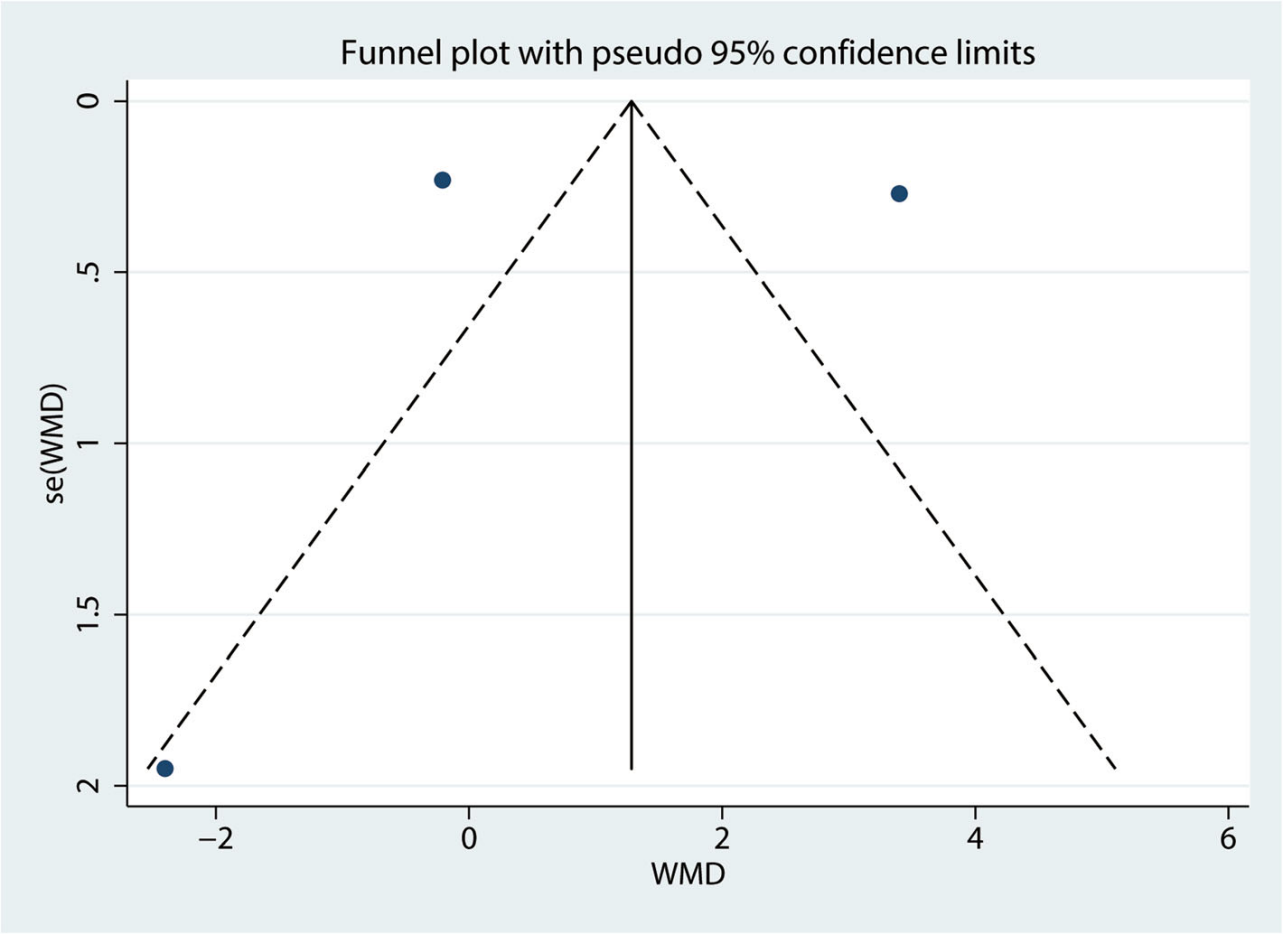
Funnel plot for comparing the DAA versus LA in terms of Harris hip score at final follow-up

**Fig. 6:**
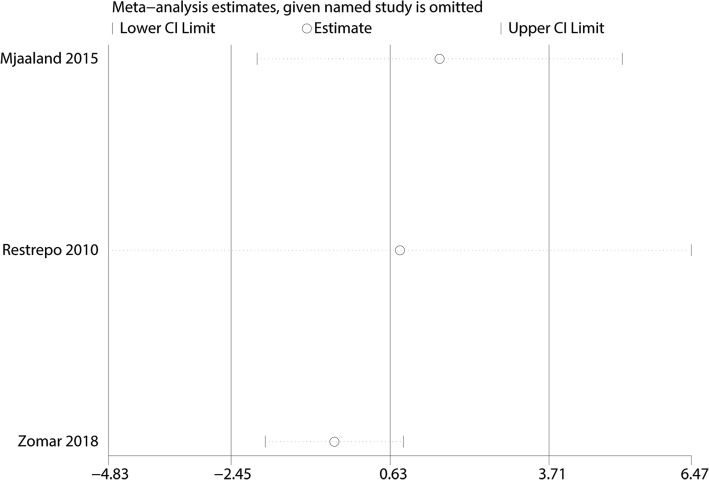
Sensitivity analysis of Harris hip score at final follow-up

A total of three studies totaling 342 THAs (DAA = 170, LA = 172) reported Harris hip scores at the final follow-up. There was no significant difference between the DAA and LA groups in terms of the Harris hip score at final follow-up (WMD = 0.63, 95% CI = − 2.54, 3.71, *P* = 0.689, Fig. 4). There was a large heterogeneity between the included studies (*I*^2^ = 98.1%); thus, we adopted a random effects model to pool the relevant data. Funnel plot analyses on the Harris hip score demonstrated symmetry, suggesting that bias was minimal (Fig. 5). Figure 6 presents the results of sensitivity analyses. The findings for the Harris hip score were consistent after omitting each study in turn.

#### VAS at 2 weeks, 6 weeks, and 12 weeks

**Fig. 7:**
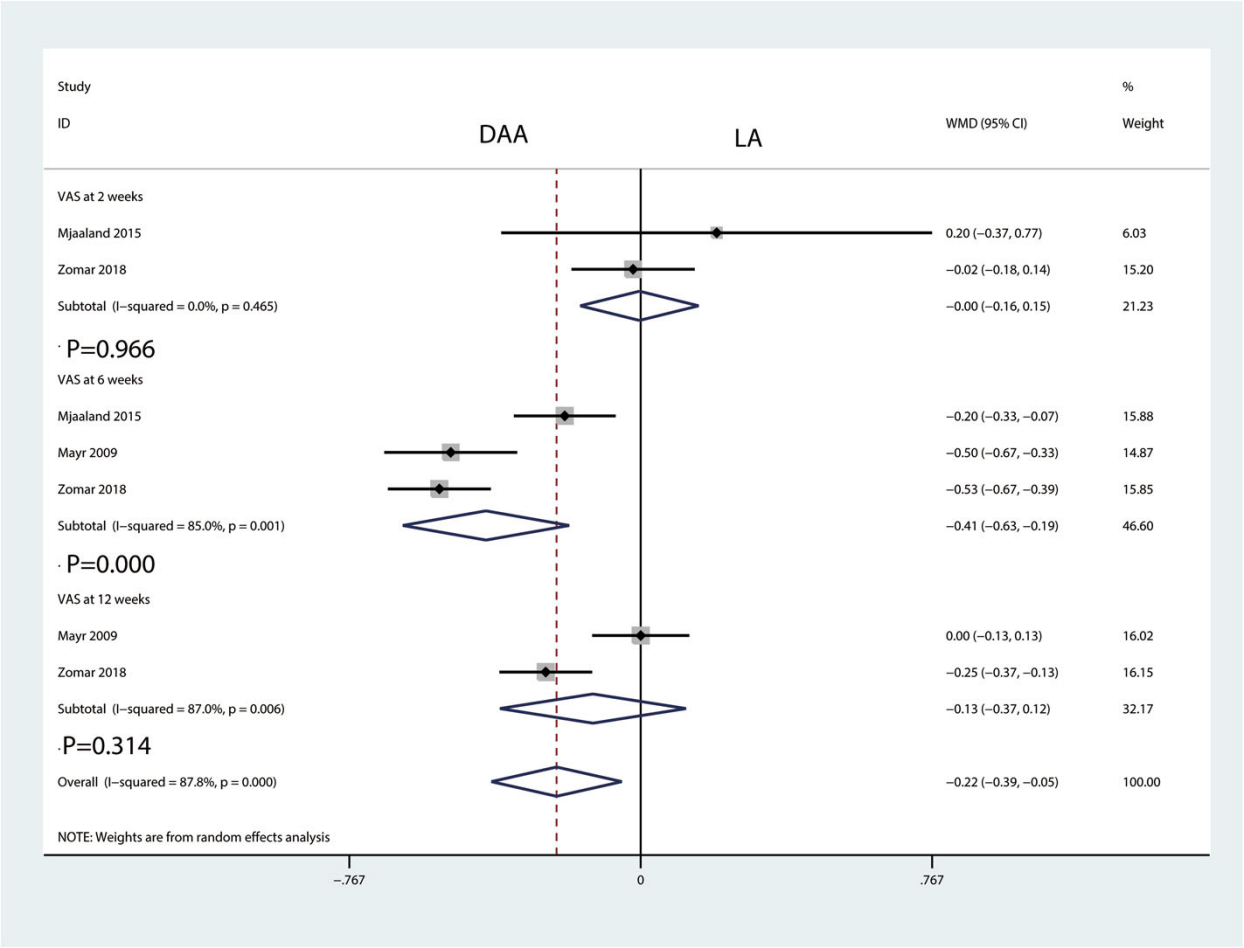
Forest plot for comparing the DAA versus LA in terms of VAS at 2 weeks, 6 weeks, and 12 weeks

There was no significant difference between the DAA and LA groups in terms of the VAS at 2 weeks (WMD = − 0.00, 95% CI − 0.16 to 0.15, *P* = 0.966, Fig. 7) and the VAS at 12 weeks (WMD = − 0.13, 95% CI − 0.37 to 0.12, *P* = 0.314, Fig. 7). Compared with the LA, the DAA was associated with a reduction in the VAS at 6 weeks (WMD = − 0.41, 95% CI − 0.63 to − 0.19, *P* = 0.000, Fig. 7).

#### Operation time

**Fig. 8:**
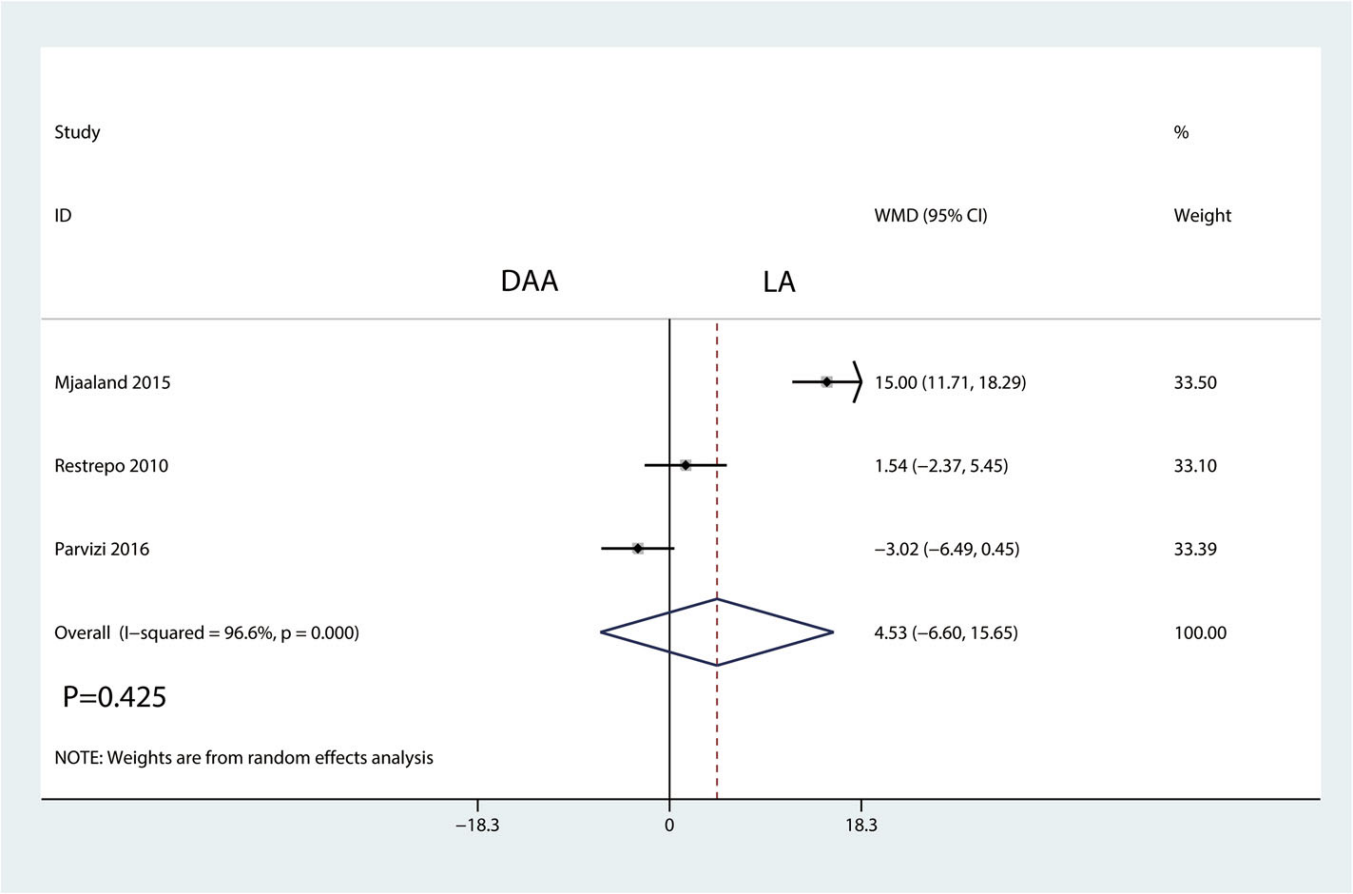
Forest plot for comparing the DAA versus LA in terms of operation time

Three trials totaling 364 patients provided data on operation time. There was no statistically significant difference between the DAA and LA groups in terms of the operation time (WMD = 4.53, 95% CI − 6.60 to 15.65, *P* = 0.425, Fig. 8).

#### Transfusion rate and total blood loss

**Fig. 9:**
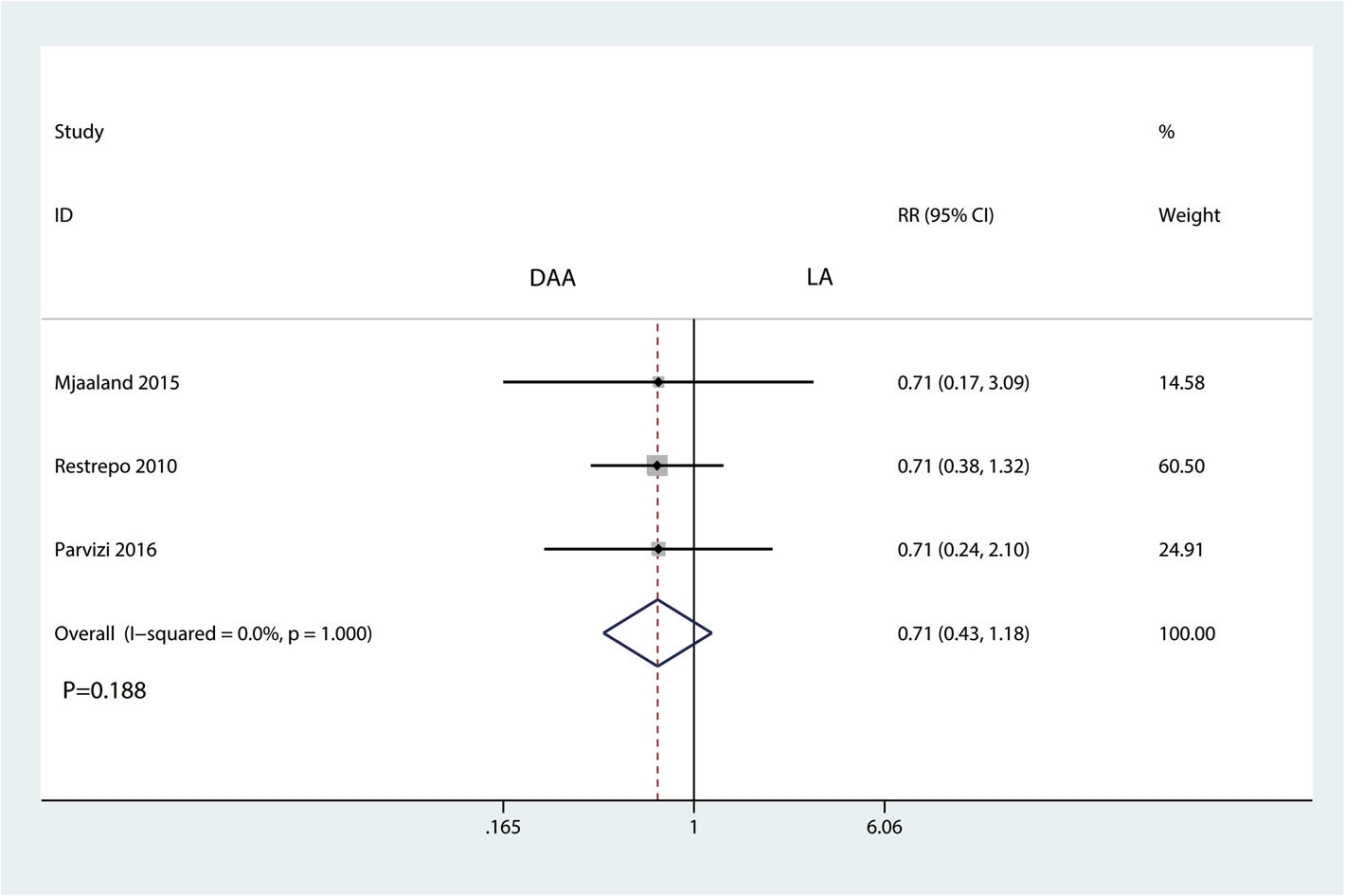
Forest plot for comparing the DAA versus LA in terms of transfusion rate

Three studies involving 364 patients provided data on the transfusion rate. Compared with the LA, the DAA had no benefit in reducing the transfusion rate after THA (RR = 0.71, 95% CI 0.43 to 1.18, *P* = 0.188, Fig. 9).

**Fig. 10:**
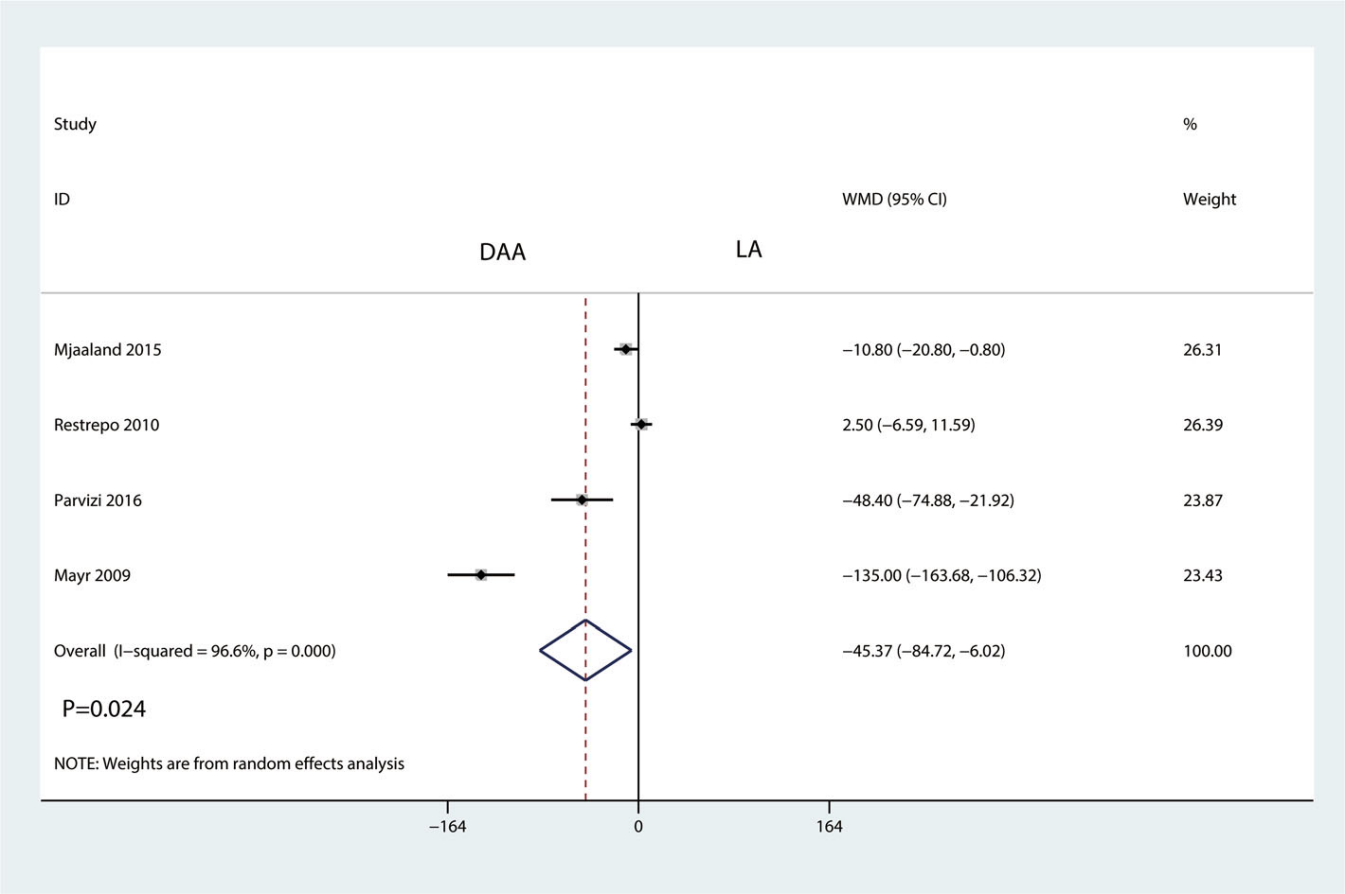
Forest plot for comparing the DAA versus LA in terms of total blood loss

Four trials totaling 397 patients provided data on total blood loss. Compared with the LA, the DAA reduced total blood loss in THA patients (WMD = − 45.73, 95% CI − 84.72 to − 6.02, *P* = 0.024, Fig. 10).

#### Length of hospital stay

**Fig. 11:**
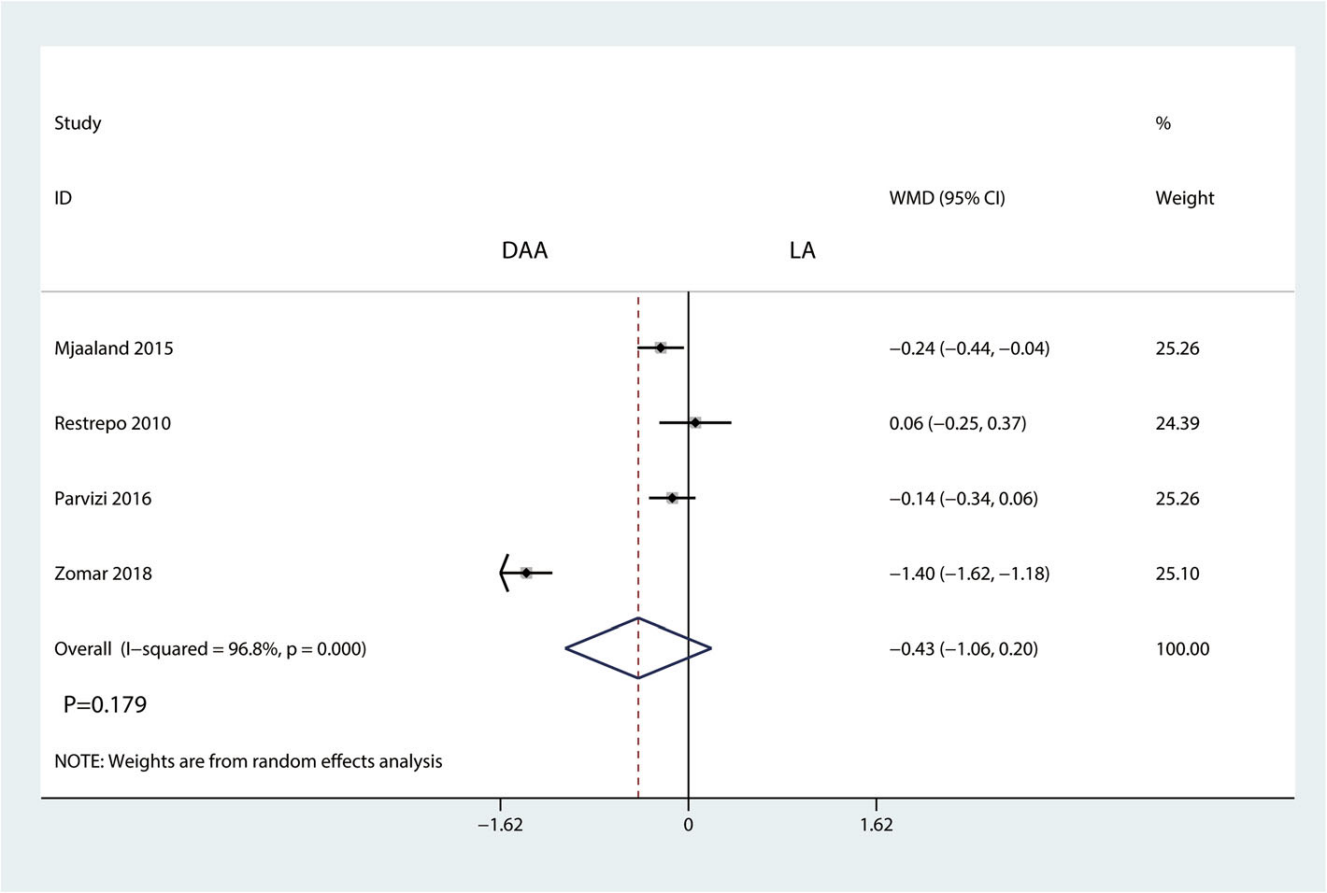
Forest plot for comparing the DAA versus LA in terms of length of hospital stay

Four trials totaling 442 patients provided data on the length of hospital stay. Compared with the LA, the DAA had no effect on reducing the length of hospital stay for THA patients (WMD = − 0.43, 95% CI − 1.06 to 0.20, *P* = 0.179, Fig. 11).

#### Complications

**Fig. 12:**
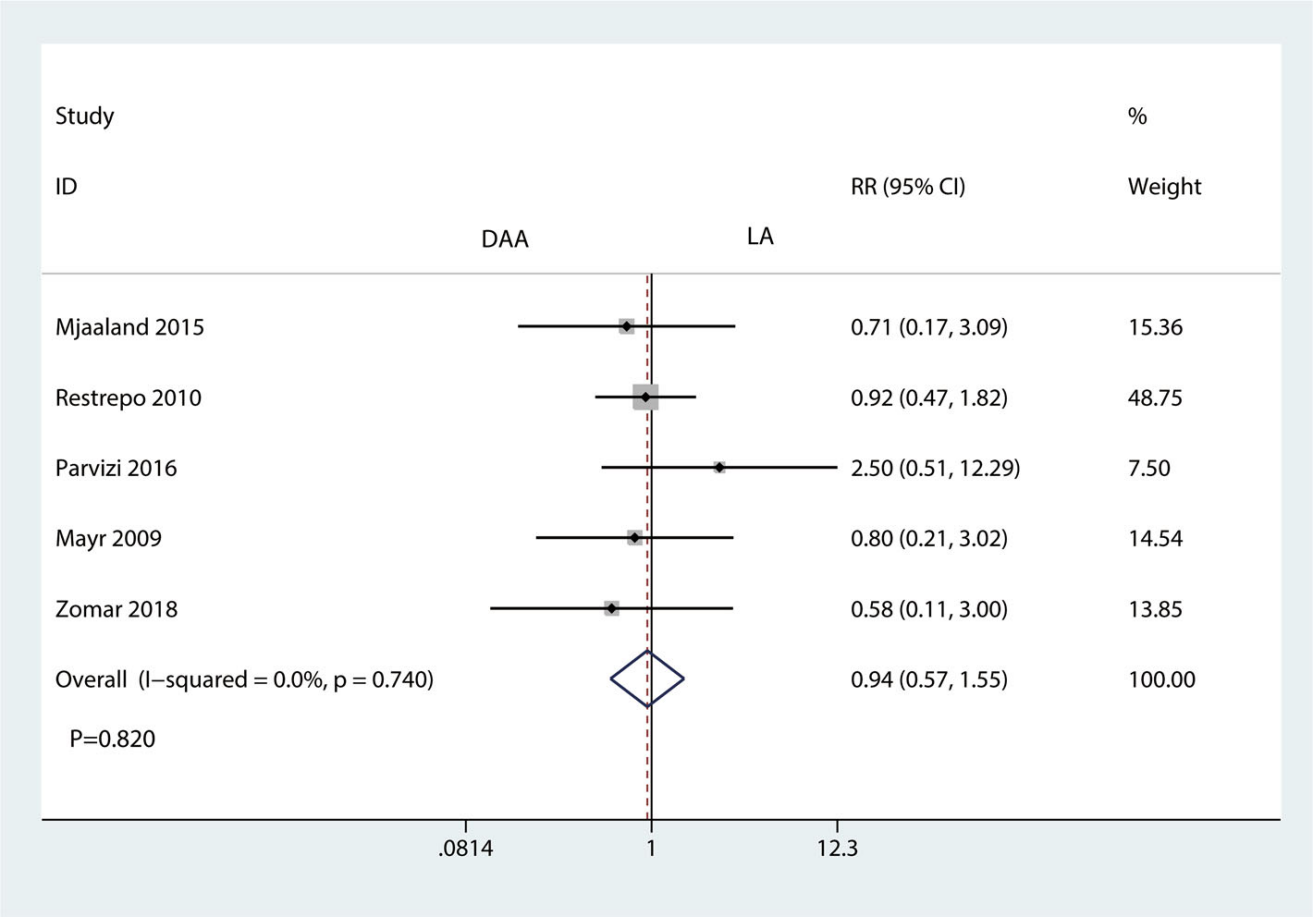
Forest plot for comparing the DAA versus LA in terms of the complications

Five trials involving 475 patients provided data on complications. The results showed that there was no significant difference in complications between the DAA and LA groups (RR = 0.94, 95% CI 0.57 to 1.55, *P* = 0.820, Fig. 12).

#### Temporal and spatial gait characteristics

**Fig. 13:**
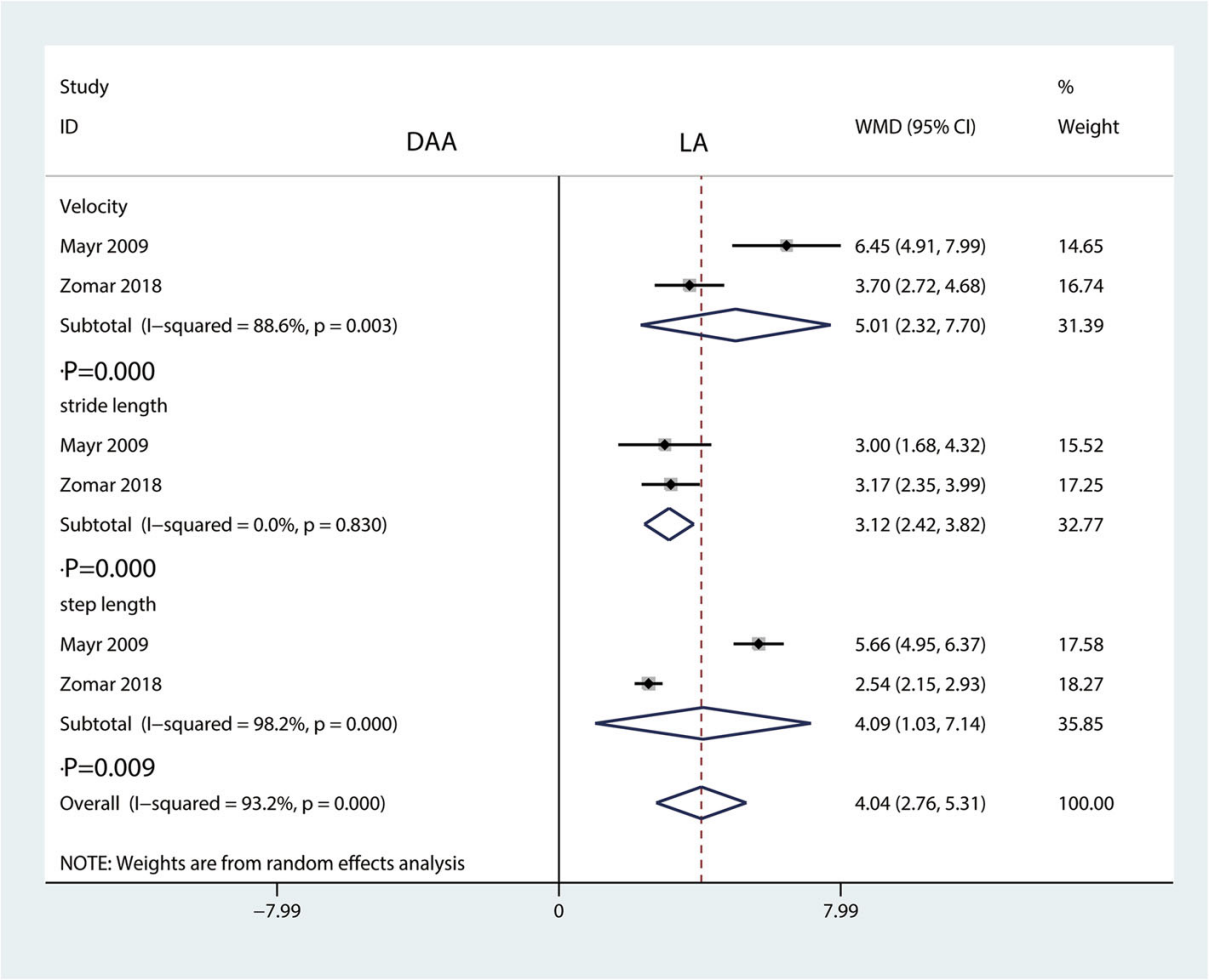
Forest plot for comparing the DAA versus LA in terms of the temporal and spatial gait characteristics (velocity, stride length, and step length)

Only two studies involving 111 patients provided data on temporal and spatial gait characteristics (velocity, stride length, and step length). Compared with the LA, the DAA was associated with an increase in walking velocity (WMD = 5.01, 95% CI 2.32 to 7.70, *P* = 0.000, Fig. 13), stride length (WMD = 3.12, 95% CI 2.42 to 3.82, *P* = 0.000, Fig. 13), and step length (WMD = 4.09, 95% CI 1.03 to 7.14, *P* = 0.009, Fig. 13).

## Discussion

### Main findings

Our systematic review and meta-analysis comprehensively and systematically reviewed the current available literature and found that in THA patients, compared with the LA, (1) the DAA significantly reduced VAS at 6 weeks and total blood loss; (2) the DAA significantly increased walking velocity, stride length, and step length in analyses of gait characteristics; and (3) the DAA had no benefit for Harris hip score, VAS at 2 weeks and 12 weeks, blood transfusion, length of hospital stay, and complications.

### Comparison with other meta-analyses

Only two meta-analyses of the DAA versus LA for THA have been published. However, differences between ours and previous ones should be noted. First, previous meta-analyses included no more than two RCTs and 106 patients that compared DAA versus LA in THA patients [11, 12]. In comparison, we included five RCTs totaling 475 patients. With the added statistical power of at least 369 patients, our current meta-analysis was the latest and the most comprehensive one. Yue et al. [11] conducted a meta-analysis that compared the DAA and LA for THA. However, in this meta-analysis, they mixed RCTs and non-RCTs in the analyses. Thus, there was a large heterogeneity in their outcomes. Second, we applied further subgroup analysis and sensitivity analysis to provide a more credible estimate. Third, we compared gait characteristics between the DAA and LA.

### Implications for clinical practice

Our meta-analysis showed that the DAA has a beneficial role in reducing postoperative pain and blood loss and increasing the function of hip joints. Therefore, the DAA might be the better approach for THA.

Furthermore, operator experience and learning curve are equally important factors. All of the included studies have reported that the operators have surpassed the learning curves; thus, the current evidence is limited, and further trials are warranted to identify differences in the learning curve between the two approaches.

First, we compared the Harris hip scores at the final follow-up. The results showed that the DAA and LA had similar hip function. Zomar et al. [17] reported that there was no significant difference between the DAA and LA groups in terms of the patient-reported functional outcomes (*P* > 0.05). However, Mirza et al. [18] revealed that the DAA encourages earlier functional recovery than LA for THA patients. We found that the DAA only had a beneficial role in reducing VAS at 6 weeks. Mjaaland et al. [10] compared pain scores between the DAA and LA groups in THA patients. The results showed that there were lower pain scores in the DAA group on all recorded days. Zomar et al. [17] found no differences in pain VAS at 2 weeks and 12 weeks postsurgery between the DAA and LA groups.

Moreover, we found that the DAA was associated with a reduction in the total blood loss compared with the LA group. The DAA is an intermuscular and internervous approach without sacrificing muscle injury. Theoretically, the DAA could significantly reduce the approach-related blood loss. Tranexamic acid was routinely administered in THA, and thus, blood loss was not an important factor for hip function. Restrepo et al. [14] estimated that blood loss was the same in the DAA and LA groups. We pooled total complications as the index for safety of these two approaches. The results showed that there was no significant difference between the groups in terms of complications.

### Strength of current meta-analysis

A major strength of our meta-analysis was that we used gait characteristics (velocity, stride length, and step length) to analyze functional outcomes. The results showed that the DAA could increase walking velocity, stride length, and step length. All of the characteristics indicated that the DAA offers significant early advantages in functional recovery compared to the LA.

### Limitations

Our meta-analysis also has several limitations: (1) only five studies (475 THAs) were included in our meta-analysis. The statistical efficacy of our results would be more reliable if more studies had been included. (2) Some studies included RCTs that lacked details of the allocation concealment and blinding methods, which may affect the quality of evidence and strength of the recommendations. (3) Follow-ups of these studies were relatively short (maximum follow-up = 2 years), and long-term follow-ups are needed to compare hip function between the DAA and LA. (4) There was substantial heterogeneity between the included outcomes. We performed subgroup analysis and sensitivity analysis to decrease the heterogeneity; however, the overall heterogeneity was not changed after subgroup analysis or after sensitivity analysis.

## Conclusion

In THA patients, compared with the LA, the DAA was associated with early functional recovery, low blood loss, and lower pain scores. There was no significant difference in Harris hip score or operation time between the DAA and LA groups. Considering the limitations of this meta-analysis, more high-quality RCTs are needed to further identify the effects of the DAA versus LA in THA patients.

## Abbreviations

CI: Confidence intervals
DAA: Direct anterior approach
LA: Lateral approach
MeSH: Medical Subject Headings
OA: Osteoarthritis
PRISMA: Preferred Reporting Items for Systematic Reviews and Meta-Analyses
RCTs: Randomized controlled trials
RR: Risk ratio
THA: Total hip arthroplasty
WMD: Weighted mean difference

## References

[1] Hojer Karlsen AP, Geisler A, Petersen PL, Mathiesen O, Dahl JB (2015). Postoperative pain treatment after total hip arthroplasty: a systematic review. Pain.

[2] Marques EM, Jones HE, Elvers KT, Pyke M, Blom AW, Beswick AD (2014). Local anaesthetic infiltration for peri-operative pain control in total hip and knee replacement: systematic review and meta-analyses of short- and long-term effectiveness. BMC Musculoskelet Disord.

[3] Karunaratne S, Duan M, Pappas E, Fritsch B, Boyle R, Gupta S, Stalley P, Horsley M, Steffens D. The effectiveness of robotic hip and knee arthroplasty on patient-reported outcomes: a systematic review and meta-analysis. Int Orthop. 2018; [Epub ahead of print]. 10.1007/s00264-018-4140-3 30219968

[4] Post ZD, Orozco F, Diaz-Ledezma C, Hozack WJ, Ong A (2014). Direct anterior approach for total hip arthroplasty: indications, technique, and results. J Am Acad Orthop Surg.

[5] Chechik O, Khashan M, Lador R, Salai M, Amar E (2013). Surgical approach and prosthesis fixation in hip arthroplasty world wide. Arch Orthop Trauma Surg.

[6] Meermans G, Konan S, Das R, Volpin A, Haddad FS (2017). The direct anterior approach in total hip arthroplasty: a systematic review of the literature. Bone Joint J.

[7] Graves SC, Dropkin BM, Keeney BJ, Lurie JD, Tomek IM (2016). Does surgical approach affect patient-reported function after primary THA?. Clin Orthop Relat Res.

[8] Petis SM, Vasarhelyi EM, Howard JL, Lanting BA (2018). Gait analysis following release of the short external rotators during an anterior approach for total hip arthroplasty. Hip Int.

[9] Petis S, Howard J, Lanting B, Jones I, Birmingham T, Vasarhelyi E (2018). Comparing the anterior, posterior and lateral approach: gait analysis in total hip arthroplasty. Can J Surg Journal canadien de chirurgie.

[10] Mjaaland KE, Kivle K, Svenningsen S, Pripp AH, Nordsletten L (2015). Comparison of markers for muscle damage, inflammation, and pain using minimally invasive direct anterior versus direct lateral approach in total hip arthroplasty: a prospective, randomized, controlled trial. J Orthop Res : official publication of the Orthopaedic Research Society.

[11] Yue C, Kang P, Pei F (2015). Comparison of direct anterior and lateral approaches in total hip arthroplasty: a systematic review and meta-analysis (PRISMA). Medicine.

[12] Putananon C, Tuchinda H, Arirachakaran A, Wongsak S, Narinsorasak T, Kongtharvonskul J (2018). Comparison of direct anterior, lateral, posterior and posterior-2 approaches in total hip arthroplasty: network meta-analysis. Eur J Orthop Surg Traumatol.

[13] Shamseer L, Moher D, Clarke M, Ghersi D, Liberati A, Petticrew M, Shekelle P, Stewart LA (2015). Preferred reporting items for systematic review and meta-analysis protocols (PRISMA-P) 2015: elaboration and explanation. BMJ (Clinical research ed).

[14] Restrepo C, Parvizi J, Pour AE, Hozack WJ (2010). Prospective randomized study of two surgical approaches for total hip arthroplasty. J Arthroplast.

[15] Parvizi J, Restrepo C, Maltenfort MG (2016). Total hip arthroplasty performed through direct anterior approach provides superior early outcome: results of a randomized, prospective study. Orthop Clin North Am.

[16] Mayr E, Nogler M, Benedetti MG, Kessler O, Reinthaler A, Krismer M, Leardini A (2009). A prospective randomized assessment of earlier functional recovery in THA patients treated by minimally invasive direct anterior approach: a gait analysis study. Clin Biomech (Bristol, Avon).

[17] Zomar BO, Bryant D, Hunter S, Howard JL, Vasarhelyi EM, Lanting BA (2018). A randomised trial comparing spatio-temporal gait parameters after total hip arthroplasty between the direct anterior and direct lateral surgical approaches. Hip Int : the journal of clinical and experimental research on hip pathology and therapy.

[18] Mirza AJ, Lombardi AV, Morris MJ, Berend KR (2014). A mini-anterior approach to the hip for total joint replacement: optimising results: improving hip joint replacement outcomes. Bone Joint J.

